# Measuring decline in white matter integrity after systemic treatment for breast cancer: omitting skeletonization enhances sensitivity

**DOI:** 10.1007/s11682-020-00319-1

**Published:** 2020-07-23

**Authors:** Yasmin Mzayek, Michiel B. de Ruiter, Hester S. A. Oldenburg, Liesbeth Reneman, Sanne B. Schagen

**Affiliations:** 1 Division of Psychosocial Research and Epidemiology, Netherlands Cancer Institute, Amsterdam, The Netherlands; 2 Brain and Cognition, Department of Psychology, University of Amsterdam, Nieuwe Achtergracht 129 B, Amsterdam, The Netherlands; 3 Department of Surgical Oncology, Netherlands Cancer Institute, Amsterdam, The Netherlands; 4 Department of Radiology, Location AMC, Amsterdam University Medical Centers, University of Amsterdam, Amsterdam, The Netherlands

**Keywords:** Cancer related cognitive impairment (CRCI), Diffusion tensor imaging (DTI), Skeletonization, Tract based spatial statistics (TBSS), Advanced Normalization Tools (ANTs)

## Abstract

Chemotherapy for non-central nervous system cancers is associated with abnormalities in brain structure and function. Diffusion tensor imaging (DTI) allows for studying *in vivo* microstructural changes in brain white matter. Tract-based spatial statistics (TBSS) is a widely used processing pipeline in which DTI data are typically normalized to a generic DTI template and then ‘skeletonized’ to compensate for misregistration effects. However, this approach greatly reduces the overall white matter volume that is subjected to statistical analysis, leading to information loss. Here, we present a re-analysis of longitudinal data previously analyzed with standard TBSS (Menning et al., BIB 2018, 324–334). For our current approach, we constructed a pipeline with an optimized registration method in Advanced Normalization Tools (ANTs) where DTI data are registered to a study-specific, high-resolution T1 template and the skeletonization step is omitted. In a head to head comparison, we show that with our novel approach breast cancer survivors who had received chemotherapy plus or minus endocrine therapy (BC + SYST, *n* = 26) showed a global decline in overall FA that was not present in breast cancer survivors who did not receive systemic therapy (BC-SYST, *n* = 23) or women without a cancer diagnosis (no cancer controls, NC, *n* = 30). With the standard TBSS approach we did not find any group differences. Moreover, voxel-based analysis for our novel pipeline showed a widespread decline in FA in the BC + SYST compared to the NC group. Interestingly, the BC-SYST group also showed a decline in FA compared to the NC group, although in much less voxels. These results were not found with the standard TBSS approach. We demonstrate that a modified processing pipeline makes DTI data more sensitive to detecting changes in white matter integrity in non-CNS cancer patients after treatment, particularly chemotherapy.

## Introduction

Cancer related cognitive impairment (CRCI) is common in cancer patients, even when the disease is not located in the central nervous system (CNS). It is particularly observed after chemotherapy (Ahles and Root 2018), can persist long after completion of treatment and may negatively affect quality of life (Ayanian and Jacobsen 2006). Various studies have reported abnormalities in brain structure and function indicative of neurotoxicity of chemotherapy (Amidi and Wu 2019; Deprez et al. 2018; Kaiser et al. 2014; Li and Caeyenberghs 2018). Brain white matter might be particularly vulnerable to chemotherapy (Dietrich et al. 2006; Gibson et al. 2019; Matsos et al. 2017).

Diffusion tensor imaging (DTI) is particularly suited to study brain white matter integrity. Fractional anisotropy (FA) and mean diffusivity (MD) (Bihan et al. 2001) are the most commonly derived measures. Generally, a decrease in FA and an increase in MD are interpreted as indicative of injury to white matter microstructure (Deprez et al. 2013).

The first longitudinal DTI study that included a pre-chemotherapy assessment confirmed earlier cross-sectional findings (Abraham et al. 2008; De Ruiter et al. 2012; Deprez et al. 2011; Kesler et al. 2015; Simó et al. 2015; Stouten-Kemperman et al. 2015a, b) and reported a widespread decrease in FA over time in breast cancer patients treated with chemotherapy compared to breast cancer patients who did not receive chemotherapy and women without cancer (Deprez et al. 2012). However, a study by our group which used a similar design could not replicate these findings (Menning et al. 2018), since we did not find a widespread decline in FA across white matter after chemotherapy.

One of the explanations for these discrepant findings might be related to different approaches for processing the MRI data, especially concerning the so-called skeletonization of white matter before submitting it to statistical analysis (Smith et al. 2006). This is a standard processing step of tract-based spatial statistics (TBSS), a common and well-established method to analyze DTI data. In this step, an FA ‘skeleton projection’ is carried out, where individual FA maps are compared to a white matter skeleton template and searched perpendicularly for maximal FA values. Only these maximal values are retained, whereas surrounding voxels are eroded. This step is used to compensate for misalignment between individual participants resulting from imperfect registration, and to gain statistical power. However, skeletonization only allows for the assessment of the effects of interest where local FA values are highest, potentially decreasing the sensitivity of the statistical analysis (Bach et al. 2014). Of note, skeletonization was not used in the study of Deprez et al. (Deprez et al. 2012).

In the current study, we aim to improve between-participant registration of DTI-derived maps to render the skeleton projection step unnecessary and potentially increase sensitivity of the DTI data analysis. We used an adapted methodology (Schwarz et al. 2014; Tustison et al. 2014) that is centered around registration algorithms collectively known as Advanced Normalization Tools (ANTs (Avants et al. 2011)). ANTs includes a diffeomorphic image registration algorithm known as Symmetric Normalization (SyN), which has high ranking performance when directly compared to many other registration tools (e.g., (Klein et al. 2009)) and has been shown to decrease misalignment and increase anatomical specificity compared to using default pipelines in TBSS (Jacobacci et al. 2019; Schwarz et al. 2014; Tustison et al. 2014).

Central to the modified pipeline is the construction of a study-specific template that is based on high-resolution T1 scans that are acquired in all participants and contain a high level of anatomical detail. DTI maps of individual participants are then registered to this template. This contrasts with the standard FSL approach we used previously, where a generic DTI template is used, and T1 scans are not included in the registration steps. As demonstrated in previous reports in other research areas, the former steps improve between-participant registration, obviating the need for skeletonization (Schwarz et al. 2014; Wintermark et al. 2014). We hypothesized that with this new approach breast cancer patients who received anthracycline-based chemotherapy plus or minus endocrine therapy (BC + SYST) would show a widespread decline in FA that would not be apparent in breast cancer patients without systemic therapy (BC-SYST) or the no-cancer controls (NC). We also provide a head to head comparison of the outcomes with the conventional processing approach published previously (Menning et al. 2018).

## Methods

### Study design

Participants were the same as reported elsewhere (Menning et al. 2018) and recruited as part of a Dutch Cancer Society funded study approved by the Institutional Review Board of the Netherlands Cancer Institute. The study was held at the Academic Medical Center and Spinoza Center for Neuroimaging, both affiliated with the University of Amsterdam. Written informed consent was acquired based on the Declaration of Helsinki and institutional guidelines.

Data were collected at two time points. For patients, baseline data were collected after surgery but before receiving adjuvant chemotherapy (M1). A follow-up session (M2) took place 6 months after the last cycle of chemotherapy for BC + SYST and at matched intervals for BC-SYST and NC. An MRI protocol was acquired including a T1-weighted three-dimensional magnetization prepared rapid gradient echo (MPRAGE) scan (TR/TE = 6.6/3.0 ms, FOV 270 × 252 mm, 170 slices, voxel size 1.05 × 1.05 × 1.20 mm, sagittal direction) and a DTI scan (32 directions, TR/TE = 8.136/94 ms, FOV 250 × 250 mm, 64 slices, voxel size 2.23 × 2.23 × 2.00 mm, b value: 1000s/mm^2^) at each time point. DTI scans were acquired in the transversal direction except for three NC whose data were collected in the sagittal direction at baseline. Data were obtained using a 3.0 T Phillips Intera full-body MRI scanner and a 3.0 T Phillips Achieva full-body MRI scanner. To optimize comparability, a SENSE 8-channel receiver head coil was used at both locations.

### Processing pipeline

The pipeline described in (Menning et al. 2018) was modified in several ways to improve registration and omit the skeletonization step of TBSS. The major differences between the pipelines are listed in Table 1.

**Table 1:** DTI processing pipelines

	Standard TBSS	Modified TBSS
Template	Generic FA template (FMRIB58_FA)	Study-specific T1 template
Registration	FNIRT	ANTs SyN
FA skeletonization	Yes	No

#### DTI preprocessing

Data were corrected for motion and eddy currents using *eddy*, a tool provided within FSL 5.0.9 (Andersson and Sotiropoulos 2016). This is an improved version of the eddy_correct which we used in (Menning et al. 2018). Then, *dtifit* (Smith et al. 2006) was used to fit the diffusion tensor at each voxel (single tensor model) to produce diffusion tensor maps. Then, *tbss_1_preproc*, the initial step of TBSS, was used on FA images in order to remove the bright halo of voxels surrounding the FA images that results from eddy current distortions. All data were visually inspected for artefacts.

#### Registration steps with ANTs

**Fig. 1:**
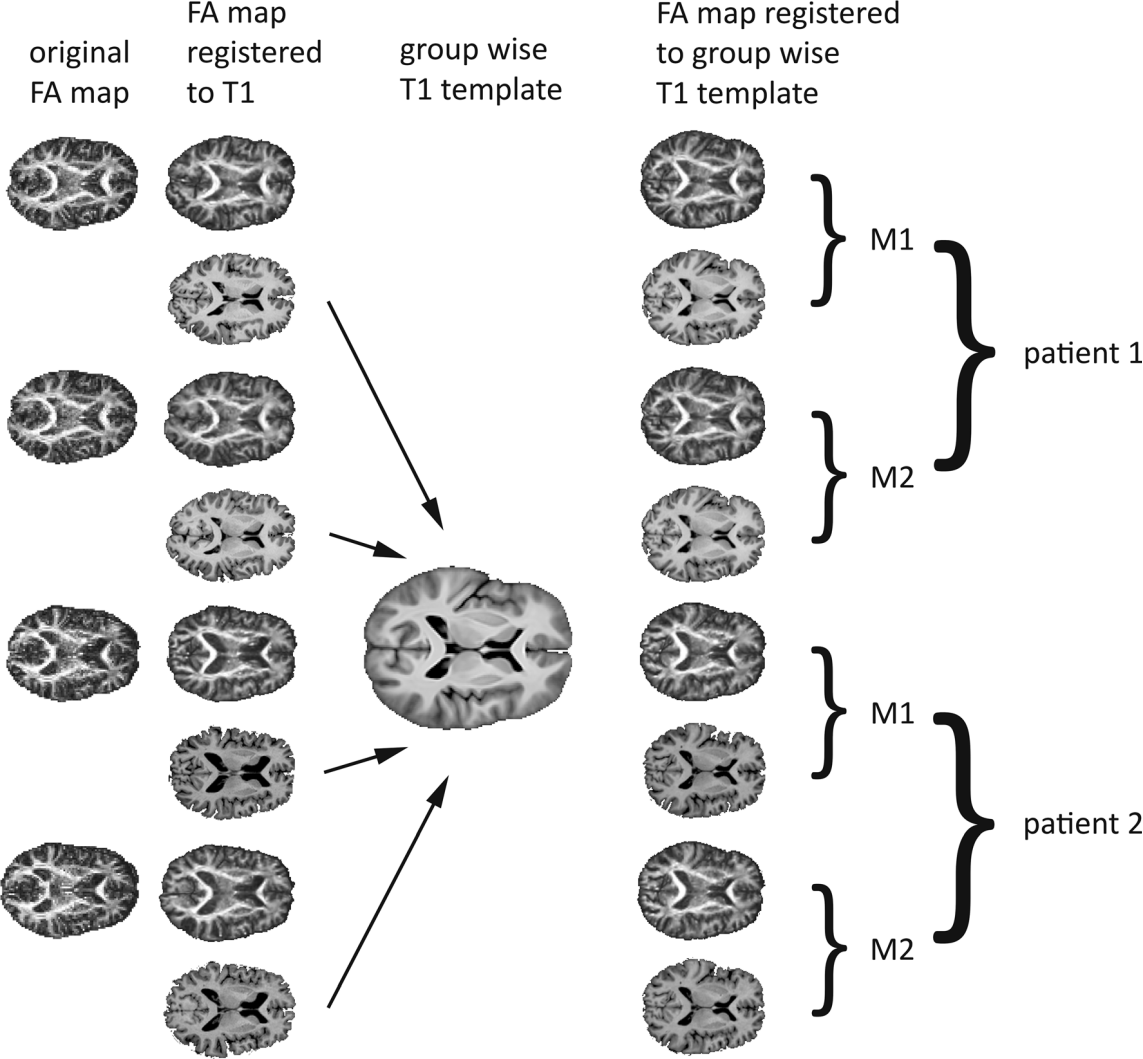
Overview of registration steps used to warp individual maps to a common space for statistical analysis

All registration steps in ANTs were carried out with ANTs v2.0 (Avants et al. 2011). In the first step, FA images from both time points (M1 and M2) were registered to their respective N4 bias corrected and skull stripped T1-weighted images using rigid, affine, and non-linear (SyN) registration. The non-linear transform algorithm was highly constrained considering this was a within subject registration. The same transformations were applied to the MD maps. In step 2, a group-wise template in native space was created using the skull stripped T1-weighted data collected for all participants at both time points, leading to a decreased image misalignment and more reliable results compared to using a generic template (Van Hecke et al. 2011) and optimally accounting for between participant differences in brain morphology by exploiting the high anatomical detail contained in the T1 scans (Wintermark et al. 2014). First, an N4 bias correction algorithm was applied to correct for image inhomogeneity (Tustison et al. 2010). The corrected images were then used to build the template using 4 iterations of rigid, affine, and non-linear (SyN) registration. This yields an unbiased template because the SyN algorithm does symmetric pair-wise mapping where mapping from one image to its target is consistent with the mapping of the target back to the image (Avants et al. 2008, 2010; Zhan et al. 2013). In step 3, the intraindividual (native FA to native T1) and interindividual (native T1 to T1 template) warping parameters were applied to the native FA and MD maps to bring them in the same space as the group-wise template (see Fig. 1). Before statistical analysis, the warped maps were blurred with a Gaussian kernel with an FWHM of 6 mm. To mainly restrict statistical analyses to white matter, a binary mask was created based on the mean of all FA maps that was thresholded at FA < 0.2.

#### Statistical analysis

First, we assessed changes in mean FA and MD by averaging across all voxels within the mask for each participant at the two time points. Group differences in mean change were analyzed with repeated-measures ANCOVA. For evaluating voxel wise changes in FA and MD, difference maps were calculated by subtracting the M1 images from the M2 images. A nonparametric general linear model using *randomise* in FSL was applied to perform paired group comparisons measuring voxel-wise differences in FA and MD change between groups. The parameters for *randomise* included 5000 permutations and threshold-free cluster-enhancement (TFCE) to correct for multiple comparisons. Scan direction was included as a covariate as DTI scans for three participants were acquired in the sagittal instead of transversal direction (Menning et al. 2018). Statistically significant outcomes were considered at a FWE corrected p < .05.

### Visualization of results

For visualization of results and anatomical reference, a mean FA map was calculated by averaging all individual warped FA maps. This map was subsequently brought to MNI space by warping it to a FA template supplied within FSL (FMRIB58_FA). The obtained warping parameters were also applied to the statistically significant results obtained with *randomise*. Localization of significant effects was inferred with the ICBM DTI-81 and the JHU white matter tractography atlases, also distributed with the FSL suite.

## Results

### Patient characteristics

Table 2 lists patient characteristics. Information about recruitment, participation, and patient demographics have been described previously in more detail (Menning et al. 2015, 2018). The final sample in this study includes 26 BC + SYST, 23 BC-SYST, and 30 NC. The groups showed no statistically significant differences in age and estimated IQ. Time between M1 and M2 was also not significantly different between groups.

**Table 2:** Patient characteristics

	BC + SYST+ (n = 26)	BC-SYST (n = 23)	NC (n = 30)	*p*
Age at M1 (years)	49.1 (8.7)	50.8 (6.5)	50.5 (8.0)	0.734
Estimated IQ (NART)	100.1 (13.6)	103.9 (13.6)	107.6 (11.4)	0.101
Education level (n, %)				
Low	0 (0)	0 (0)	0 (0)	0.091
Middle	4 (15)	3 (13)	0 (0)	
High	22 (85)	20 (87)	30 (100)	
Interval M1 – M2 (days)	332 (70)	342 (33)	363 (59)	0.119
Scan location at M2 (n)	18/8	20/3	15/15	0.017
Postmenopausal (n, %)				
M1	10 (38)	12 (52)	16 (53)	0.484
M2	26 (100)	13 (57)	16 (53)	0.001
Lifetime estrogen exposure (yrs)	31.4 (6.0)	33.9 (6.0)	32.6 (6.2)	0.356
Medication use at M2 (n, %)				
Anti-diabetic		1 (4)	1 (3)	0.588
Cardiovascular	3 (12)	5 (22)	7 (23)	0.492
Psychotropic	6 (24)	1 (4)	3 (10)	0.124
Breast cancer stage (n, %)				
0	0 (0)	12 (52)		0.001*
1	14 (54)	11 (48)		
2	11 (42)	0 (0)		
3	1 (4)	0 (0)		
Surgery (n, %)				0.790
WLE	16 (62)	15 (65)		
Ablatio	10 (39)	8 (35)		
Radiotherapy (n, %)	21 (81)	15 (65)		0.218
Tamoxifen (n, %)	17 (65)	NA		
Chemotherapy (n, %)				
AC^1^	3 (12)			
AC-docetaxel^2^	17 (65)			
AC-paclitaxel^3^	3 (12)			
FEC^4^	3 (12)			
Days since chemotherapy	201 (69)			

Values indicate mean ± SD unless indicated otherwise. BC + SYST, BC patients who received systemic treatment; BC-SYST, BC patients not requiring systemic treatment; NC, no-cancer controls. Scan location at M2 depicts number of participants at the two scan locations. Lifetime estrogen exposure was calculated by subtracting age at menarche from the age at menopause or current age, for each pregnancy an additional 0.75 year was subtracted (Schilder et al. 2010). WLE = wide local excision; Ablatio = breast amputation. AC = doxorubicin (Adriamycin), cyclophosphamide; FEC = 5-fluorouracil, epirubicin, cyclophosphamide. ^1^4 or 6 cycles; ^2^3 or 6 cycles; ^3^4 cycles AC followed by 4 or 12 cycles of paclitaxel; ^4^3 or 6 cycles. ANOVA or chi-square was used for statistical testing. * p < .001

### Statistical results

**Fig. 2:**
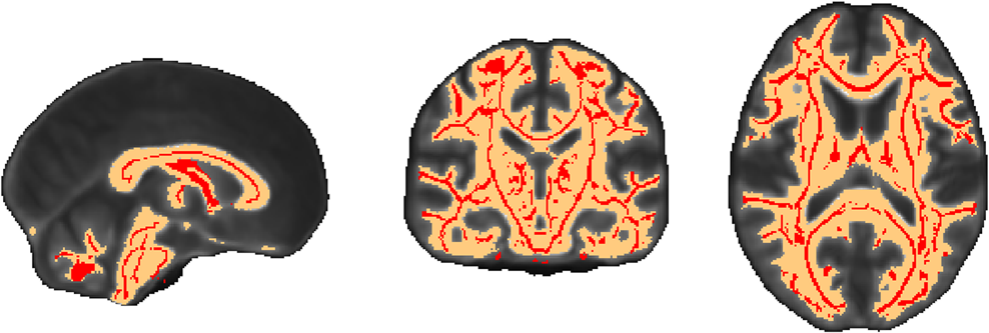
White matter skeleton mask (in red) used in Menning et al. (2018) and white matter mask used in present analysis (in orange). White matter mask was warped from native space to standard MNI space (similar to FA skeleton) for ease of viewing

Compared to the FA skeleton used in standard TBSS with a volume of 125 ml, masked images in the improved pipeline included a considerably larger volume of 688 ml (see Fig. 2).

#### Average FA and MD

##### Modified TBSS

**Fig. 3:**
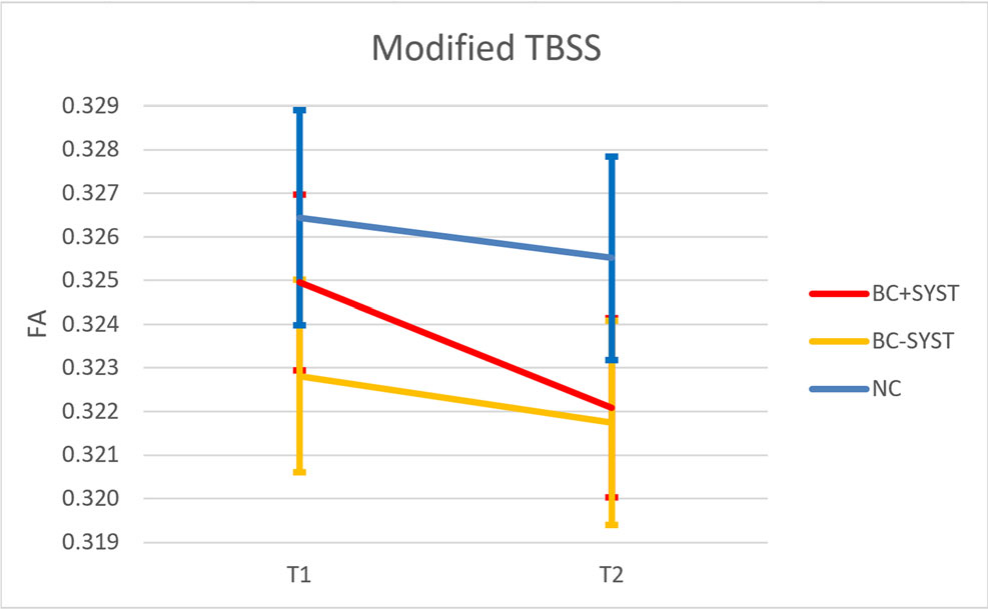
Changes in overall mean FA across white matter. FA significantly decreases for BC + SYST (breast cancer patients exposed to chemotherapy ± endocrine therapy), not for BC-SYST (breast cancer patients not exposed to systemic treatment) or NC (no cancer controls without a cancer diagnosis). See text for statistics

For overall whole brain FA, a significant main effect of time (*F*(1, 75) = 14.302, *p* < .001) indicated a decrease in FA from M1 to M2. A significant Group x Time interaction (*F*(1, 75) = 3.696, *p* = .029) indicated that the decline in FA differed per group. Paired sampled t tests per group showed a significant decline in FA in the BC + SYST group: *t*(25) = 4.357, *p* < .001. No significant changes over time were found for the BC-SYST group (*t*(22) = 1.628, *p* = .12) or the NC group (*t*29) = 1.350, *p* = .19. No main effect of Group was found for FA (*F*(1, 75) < 1, NS) (See Table 3; Fig. 3).

**Table 3:** Overall mean DTI values within white matter mask (modified TBSS) and white matter skeleton (standard TBSS). SD is in parentheses

		M1			M2		
		BC + SYST (n = 26)	BC-SYST (n = 23)	NC (n = 30)	BC + SYST (n = 26)	BC-SYST (n = 23)	NC (n = 30)
Modified TBSS	FA	0.325 (0.010)	0.323 (0.011)	0.326 (0.013)	0.322 (0.011)	0.322 (0.011)	0.326 (0.013)
	MD	0.901 (0.030)	0.899 (0.030)	0.895 (0.029)	0.902 (0.033)	0.903 (0.034)	0.897 (0.029)
Standard TBSS	FA	0.440 (0.016)	0.437 (0.015)	0.445 (0.020)	0.437 (0.013)	0.436 (0.015)	0.441 (0.018)
	MD	0.736 (0.018)	0.739 (0.018)	0.727 (0.025)	0.737 (0.017)	0.740 (0.021)	0.732 (0.022)

For MD, a marginally significant main effect of time suggested an increase in MD from M1 to M2 (*F*(1, 75) = 3.060, *p* = .084). No significant effects for Time X Group or Group were found (all *F*s < 1, NS).

##### Standard TBSS

For the skeletonized data, ANCOVA on the FA data revealed significant main effect of time (*F*(1, 75) = 19.428, *p* < .001) indicating a decrease in FA from M1 to M2. No other significant effects were found for FA or MD (all Fs < 2, NS) (Menning et al. 2018).

#### Voxel-wise modified TBSS analysis

**Fig. 4:**
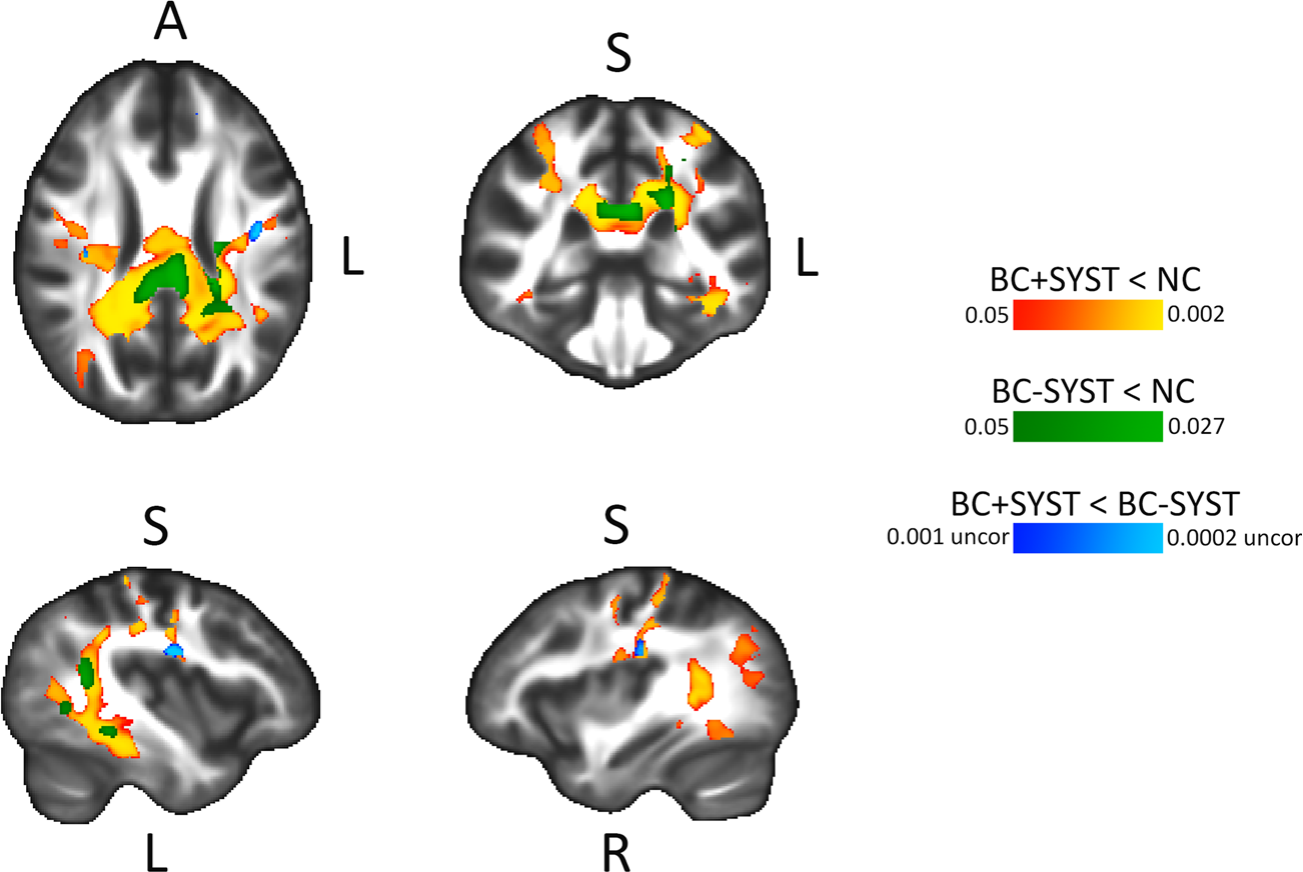
Results from voxel-wise modified TBSS analysis of FA maps. Effects show a decrease in FA from M1 to M2. BC + SYST < NC: more decrease in patients exposed to chemotherapy ± endocrine treatment vs. no cancer controls; BC-SYST < NC: more decrease in cancer patients not exposed to systemic treatment vs. no cancer controls; BC + SYST < BC-SYST: more decrease in patients exposed to chemotherapy ± endocrine treatment vs. cancer patients not exposed to systemic treatment. Analyses are TFCE corrected at p < .05 except for the BC + SYST < BC-SYST contrast. Significant effects are overlaid on the average FA of all participants. Statistical analyses were performed in native space. For visualization and anatomical reference, results were warped to MNI space

Table 4 shows the results of the whole brain voxel-wise analysis. The pairwise group differences show a significant decrease in FA in the BC + SYST group vs. the NC group in a large number of voxels. Figure 4 shows a widespread bilateral distribution of affected white matter regions with a posterior dominance. Involved tracts include bilateral superior longitudinal fasciculus (SLF), bilateral sagittal stratum, bilateral posterior thalamic radiation, bilateral cingulum, bilateral corticospinal tract, bilateral corona radiata, splenium and body of the corpus callosum. For the BC-SYST vs. NC contrast, we also found a decrease in FA, albeit in a much lower number of voxels. Affected regions included left SLF, left corticospinal tract, left corona radiata, splenium of corpus callosum. At our predetermined threshold of p < .05 (TFCE, FWE corrected) we did not find significant group differences in decline in FA between BC + SYST and BC-SYST. When inspecting the results at a p < .001 uncorrected, however, we observed some small foci including bilateral SLF, indicating a steeper decrease of FA in these regions in the BC + SYST than the BC-SYST group. Few significant differences were found with regard to MD. The BC + SYST vs. NC comparison showed a significant decrease in MD in BC + SYST relative to NC in the body of the corpus callosum. When directly comparing BC + SYST vs. BC-SYST at an uncorrected p < .001 we found a steeper decrease in MD for BC + SYST than BC-SYST in the left sagittal stratum and left retrolenticular part of the internal capsule.

**Table 4:** Voxel-wise group analyses. Total volume of significantly different voxels between groups (in ml, percentage of total white matter in parentheses)

Modified TBSS		BC + SYST < NC	BC-SYST < NC	BC + SYST < BC-SYST*
	FA	44.4 (6.5)	3.9 (0.6)	2.5 (0.4)
	MD	0.7 (0.1)	0 (0)	1.0 (0.1)
		BC + SYST > NC	BC-SYST > NC	BC + SYST > BC-SYST*
	FA	0.9 (0.1)	0	0
	MD	0	0	0.4 (0.1)
Standard TBSS		BC + SYST < NC	BC-SYST < NC	BC + SYST < BC-SYST
	FA	0	0	0
	MD	0	0	0
		BC + SYST > NC	BC-SYST > NC	BC + SYST > BC-SYST
	FA	0	0	0
	MD	0	0	0

Results from voxel-wise TBSS analyses. Shown is total volume in ml (percentage of total white matter volume in parentheses) of significantly different voxels in pairwise group comparisons performed in randomise on DTI difference maps (M2 – M1). BC + SYST < NC: more decrease in patients exposed to chemotherapy ± endocrine treatment vs. no cancer controls; BC-SYST < NC: more decrease in cancer patients not exposed to systemic treatment vs. no cancer controls; BC + SYST < BC-SYST: more decrease in patients exposed to chemotherapy ± endocrine treatment vs. cancer patients not exposed to systemic treatment. Analyses are FWE corrected at *p* < .05 except for the BC + SYST < BC-SYST and BC + SYST > BC-SYST contrasts (*thresholded at* p *< .001 uncorrected)

## Discussion

As hypothesized, applying a modified TBSS pipeline to our DTI data tremendously improved the sensitivity to detect chemotherapy-associated decline in white matter microstructure in breast cancer patients. Using improved registration techniques compared to the standard TBSS preprocessing pipeline (see Bach et al. 2014; Jacobacci et al. 2019; Schwarz et al. 2014; Tustison et al. 2014; Wintermark et al. 2014) allowed us to skip the skeletonization step and retain much more white matter that could be submitted to statistical analysis (688 ml instead of 125 ml). Our first main finding was that the overall mean FA (averaged across white matter) showed a significant decline in the BC + SYST group that was absent in the two other groups. To our knowledge, this is the first longitudinal study that shows a global decline in white matter integrity that is specifically associated with systemic cancer treatment (chemotherapy that was followed by endocrine treatment in 65% of the patients) in non-CNS cancer patients.

Moreover, our voxel-wise analysis showed a widespread reduction in FA from baseline to 6 months after chemotherapy when compared to the NC group (women without a cancer diagnosis). Affected white matter regions were largely bilaterally and somewhat posteriorly localized, including SLF, posterior thalamic radiation, cingulum, corticospinal tract, corona radiata and the splenium and body of the corpus callosum. These regions overlap with those previously reported in the longitudinal study by Deprez and coworkers (Deprez et al. 2012) who also did not skeletonize their data and used in-house developed registration algorithms to warp individual FA maps to a study-specific FA template (T1 scans were not used). A follow-up study to Deprez et al. (Billiet et al. 2018) observed a return to baseline values of FA 3 to 4 years after chemotherapy, suggesting recovery of white matter integrity after initial injury. Application of a skeletonization step might also explain the absence of voxel-based decline in FA in other studies in the field of CRCI (Chen et al. 2019; Correa et al. 2016; Mo et al. 2017).

Interestingly, the BC-SYST group also showed a decline in white matter integrity compared to the NC group, albeit to a much lesser extent: the total number of voxels that showed a significant decline was only a fraction compared to that observed in the BC + SYST group. Nevertheless, this is an intriguing finding that was not reported by Deprez et al., perhaps because they did not directly compare DTI changes between groups. The finding of a decline in FA in patients not exposed to chemotherapy suggests that systemic treatment is not the only factor involved in abnormalities in brain structure and function and underscores the importance of investigating the growing group of cancer survivors who did not receive systemic cancer treatments (Ahles and Root 2018). Several factors might have contributed to these findings, for instance effects of cancer itself, comorbid disease, other treatment modalities (surgery, radiotherapy) or psychosocial variables (Ahles and Root 2018). The currently observed decline in FA in the BC-SYST group explains why we did not find significant group differences in FA for BC + SYST at our predetermined stringent statistical threshold in a direct comparison with BC-SYST. When lowering this threshold, however, we observed some small foci including bilateral SLF, indicating a steeper decrease in FA in the BC + SYST than the BC-SYST group. Inclusion of a larger sample would possibly have yielded sufficient statistical power to demonstrate solid group differences in the BC + SYST vs. the BC-SYST group comparison.

The current study has several limitations. First, improvements in our processing pipeline were made on multiple levels at the same time. We did not evaluate which modification contributed the most to increasing the sensitivity of the analysis. Changes within the FSL software suite might also have contributed to increased sensitivity (the eddy current correction tool *eddy* that replaced *eddy_correct* showed superior performance compared to its predecessor (Andersson and Sotiropoulos 2016). Second, the use of a relatively lenient white matter mask increases the chance of observing FA changes outside white matter. Inspection of the data, however, did not give us the impression this was the case. Third, endocrine therapy might have had independent detrimental effects on white matter (Zwart et al. 2015). Fourth, in addition to white matter changes being due to chemotherapy neurotoxicity, these changes might also be (party) attributable to chemotherapy induced amenorrhea (Conroy et al. 2013; Peper et al. 2011). Finally, there is no gold standard to compare the DTI results with, that confirm the observed effects with the newer pipeline. So we formally cannot state that our new pipeline is more sensitive than the original pipeline.

Major strengths of our study include the longitudinal design and the inclusion of a double control group to separate effects of systemic treatment from DTI changes also apparent in other breast cancer patients. We describe a modified processing pipeline for DTI analyses with increased sensitivity to detect neurotoxicity of cancer treatment on white matter, particularly chemotherapy. To our knowledge, this is the first study to use this approach to evaluate the side effects of non-CNS cancer on the brain. The implementation of this pipeline should be feasible for other research groups as well.

### Conclusions

Our present study underscores the importance of comparing different processing pipelines in neuroimaging research. This might reveal that the results reported in different studies might altogether be more in agreement than what has been previously concluded. The findings from the present study demonstrate that a modified processing pipeline, in which the skeletonization step is omitted after improved registration, makes DTI data more sensitive to measure a decrease in brain white matter integrity in non-CNS cancer patients after treatment, particularly chemotherapy.
